# MIG and the Regulatory Cytokines IL-10 and TGF-β1 Correlate with Malaria Vaccine Immunogenicity and Efficacy

**DOI:** 10.1371/journal.pone.0012557

**Published:** 2010-09-03

**Authors:** Susanna J. Dunachie, Tamara Berthoud, Sheila M. Keating, Adrian V. S. Hill, Helen A. Fletcher

**Affiliations:** Centre for Clinical Vaccinology and Tropical Medicine, The Jenner Institute, University of Oxford, Oxford, United Kingdom; University of California Los Angeles, United States of America

## Abstract

Malaria remains one of the world's greatest killers and a vaccine is urgently required. There are no established correlates of protection against malaria either for natural immunity to the disease or for immunity conferred by candidate malaria vaccines. The RTS,S/AS02A vaccine offers significant partial efficacy against malaria.

mRNA expression of five key cytokines interferon-gamma (IFN-γ), monokine induced by gamma (MIG), interleukin-10 (IL-10), transforming growth factor-β (TGF-β) and forkhead box P3 (FoxP3) in peripheral blood mononuclear cells were measured by real-time RT-PCR before and after vaccination with RTS,S/AS02A and Modified Vaccinia virus Ankara encoding the circumsporozoite protein (MVA-CS) in healthy malaria-naïve adult volunteers. The only significant change was in IFN-γ mRNA expression, which was increased seven days after vaccination (*P* = 0.04). Expression of MIG mRNA seven days after vaccination correlated inversely with time to detection of parasites by blood film in an experimental sporozoite challenge (r = 0.94 *P* = 0.005). An inverse relationship was seen between both TGF-β1 and IL-10 mRNA at baseline and the anti-circumsporozoite IgG antibody response (r = −0.644 *P* = 0.022 and r = −0.554 *P* = 0.031 respectively). This study demonstrates the potential for MIG expression as a correlate of protection against malaria. Baseline levels of the regulatory cytokines TGF-β and IL-10 inversely correlated with antibody levels post vaccination and warrant further studies to improve understanding of individual differences in response to vaccination.

## Introduction

A vaccine for malaria is urgently required, but clear correlates of immunity against malaria have not been established. A better understanding of immune markers induced by candidate malaria vaccines would greatly enhance vaccine development, immunogenicity monitoring and estimation of vaccine efficacy in the field. Neither IFN-γ secretion, nor antibody levels correlate consistently with protection from malaria [1]. Many studies of T-cell effector function in mice [2], malaria-exposed humans [3] and vaccinated malaria-naïve populations [4] have underlined the complexity and diversity of T-cell immunity. Antibodies, CD8+ T cells, CD4+ T cells, IFN-γ, IL-12 and nitric oxide (NO) have all been implicated as critical effectors in protection against pre-erythrocytic stage malaria [5]. There is increasing evidence that, in addition to antibodies, protection from blood-stage malaria is determined by the balance of pro and anti-inflammatory immune responses induced by the parasite [3], [6]–[9].

The only candidate vaccine to demonstrate reproducible efficacy in the field is RTS,S, a pre-erythrocytic stage vaccine based on the *P. falciparum* circumsporozoite (CS) protein and administered in either the proprietary adjuvant AS02A (RTS,S/AS02A), or more recently in adjuvant AS01E (RTS,S/AS01E). RTS,S/AS02A induces strong IgG antibody responses to the NANP repeat region of the circumsporozoite antigen, as well as some CD4+ T-cell responses [4], [10]. This vaccine has been shown to confer protection against clinical malaria in a significant proportion of healthy non-immune U.S. adults in challenge studies [11], and partial protection in field studies [12]–[15] More recently a phase IIb trial of RTS,S administered in the adjuvant AS01E in Kenyan children aged 5–17 months reported an efficacy against clinical malaria of 53% [16] for eight months of follow-up and phase III trials are underway across Africa.

A clinical trial conducted in the UK [17] aimed to enhance the immunogenicity of RTS,S/AS02A alone by combining it in a prime-boost strategy with MVA that encoded the circumsporozoite (CS) protein. T-cell responses as measured by IFN-γ *ex vivo* ELISPOT assays were induced, but the responses were low to moderate, with heterologous boosting yielding only small increments in T-cell immunogenicity and no enhancement in antibody responses. No increase in protection against sporozoite challenge compared to RTS,S/AS02A alone was seen [16]. Nevertheless, as a total of four volunteers, two from each arm of the study, developed sterile protection this trial provided an opportunity to monitor responses to the circumsporozoite antigen before and after vaccination with RTS,S/AS02A in an effort to identify immune correlates of protection.

Our group has previously reported an association between the up-regulation of TGF-β1, FoxP3 and the generation of Treg cells along with faster rates of parasitic growth in subjects infected with *P. falciparum* [8]. We have also demonstrated that MIG (CXCL9), as a marker of bioactive IFN-γ, is useful for measuring vaccine induced pro-inflammatory immune responses [18] in line with a previous report [19].We hypothesised that levels of anti-inflammatory and pro-inflammatory cytokines may be associated with vaccine efficacy and we have used real time RT-PCR to monitor changes in TGF-β1, FoxP3, IL-10, IFN-γ and MIG in malaria-naïve adults receiving the candidate malaria vaccines RTS,S/AS02A and MVA-CS in a clinical trial. Although the number of subjects included in the clinical trial with RTS,S/AS02A and MVA-CS was small, such exploratory studies with real time RT-PCR may help to guide the selection of immune markers for analysis in larger efficacy trials.

## Results

### Vaccine induced changes in gene expression and correlation with protection from malaria challenge

In this trial subjects received two doses of the RTS,S/AS02A (R vaccine) vaccine (R vaccine) (GSK Biologicals, Rixensart, Belgium) and one dose of MVA-CS (M vaccine) (Oxford University, Oxford, UK). 28 days after the final immunisation the efficacy of the vaccine schedule (either MRR or RMM) was assessed in twelve of the volunteers by experimental sporozoite challenge.

**Figure 1:**
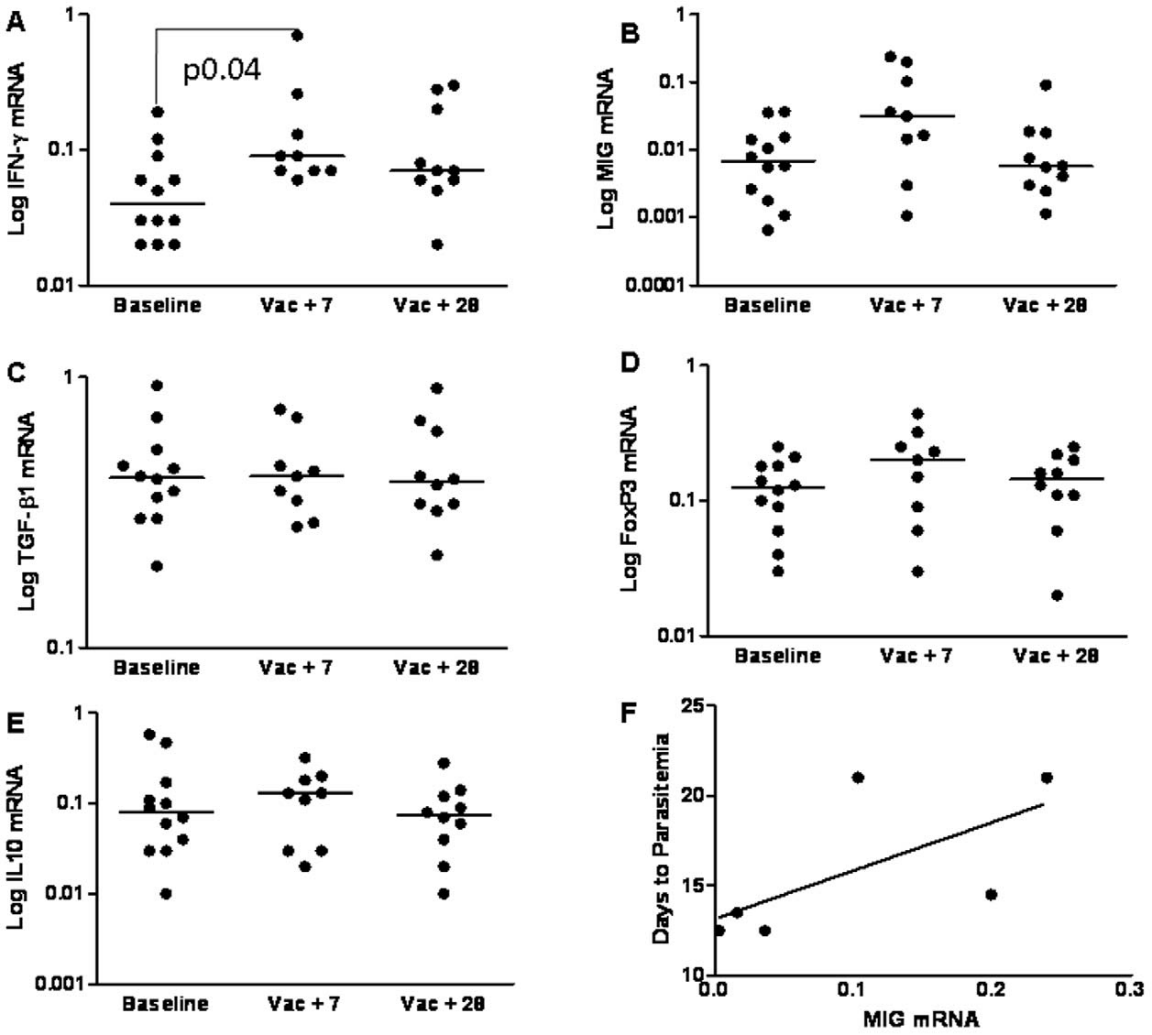
The Expression of Cytokines in CS-stimulated Cells Before and After Vaccination. Expression of IFN-γ, MIG, TGF-β1, FoxP3 and IL-10 was measured by real-time RT-PCR in total PBMCs following 12 hour culture with the vaccine antigen CS in subjects who received vaccination with RTS,S/AS02A and MVA-CS. Results are expressed as copy number relative to the housekeeping gene HPRT. Median values (9 subjects) are shown. Vac+7 =  seven days after the final vaccination, Vac+28 = 28 days after the final vaccination, each subject received two doses of RTS,S/AS02A and one dose of MVA-CS. A) IFN-g mRNA, B) MIG mRNA, C) TGF-β1 mRNA, D) FoxP3 mRNA, E) IL-10 mRNA and F) Correlation of MIG mRNA expression at day 7 with days to parasitemia following sporozoite challenge, n = 6–12.

**Figure 2:**
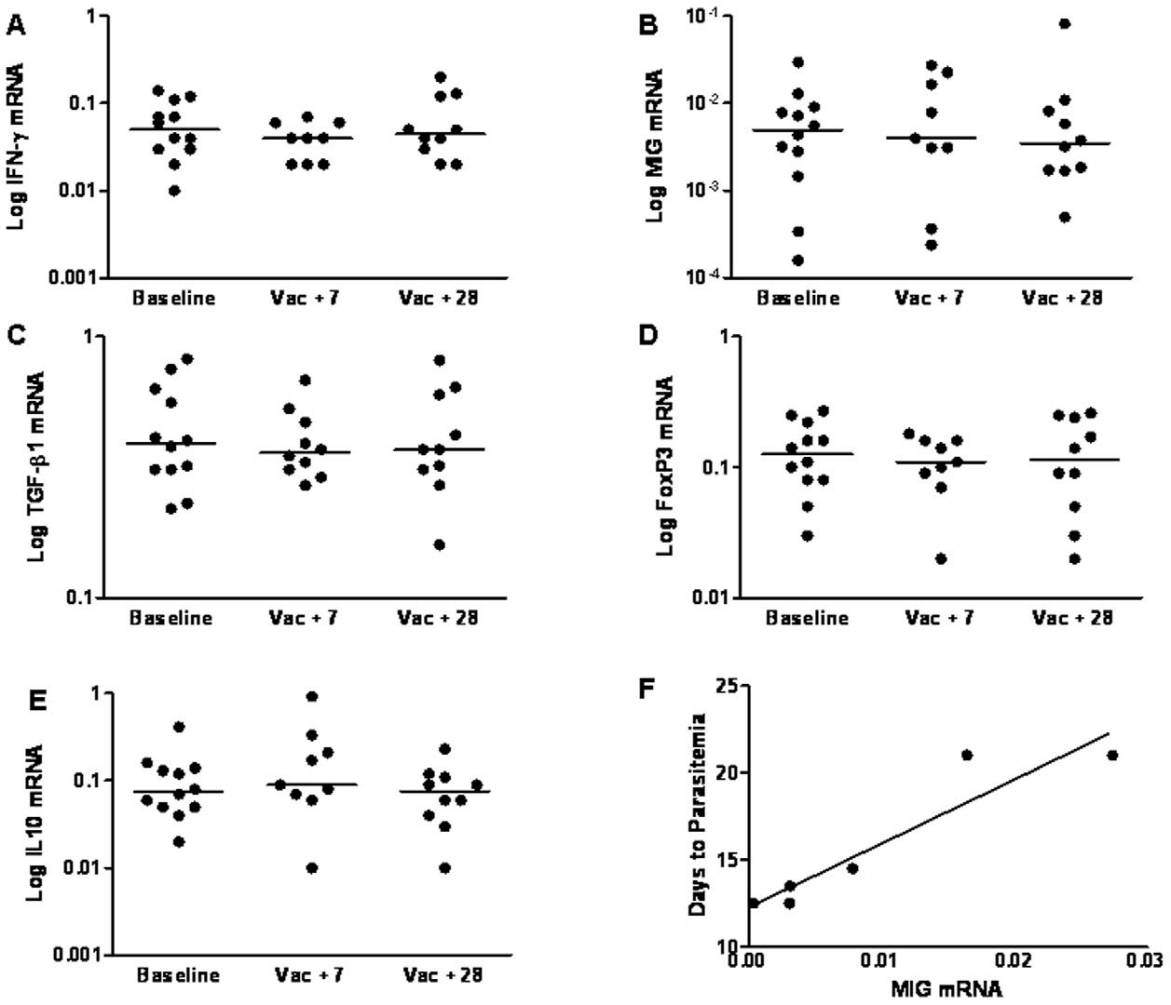
The Expression of Cytokines in Unstimulated Cells Before and After Vaccination. Expression of IFN-γ, MIG, TGF-β1, FoxP3 and IL-10 was measured by real-time RT-PCR in total PBMCs following 12 hour culture in media only in subjects who received vaccination with RTS,S/AS02A and MVA-CS. Results are expressed as copy number relative to the housekeeping gene HPRT. Median values (6–12 subjects) are shown. Vac+7 =  seven days after the final vaccination, Vac+28 = 28 days after the final vaccination, each subject received two doses of RTS,S/AS02A and one dose of MVA-CS. A) IFN-g mRNA, B) MIG mRNA, C) TGF-β1 mRNA, D) FoxP3 mRNA, E) IL-10 mRNA and F) Correlation of MIG mRNA expression at day 7 with days to parasitemia following sporozoite challenge.

Gene expression studies were performed using cryopreserved samples from subjects before and after vaccination (Day 0, the day of first vaccination, and 7 and 28 days after the final vaccination). For each cytokine studied expression levels relative to the housekeeping gene HPRT were determined for both CS stimulated (Figure 1) and unstimulated PBMCs (Figure 2), and the fold change in expression level in the CS-stimulated cells compared to the unstimulated cells at each timepoint determined (Table 1).

**Table 1:** Foldchange of Gene Expression at Each Timepoint in CS-Stimulated Cells Compared to Unstimulated Cells.

	Day 0	Vac+7	Vac+28
**IFN-γ median foldchange (range)**	0.9 (0.4–1.8) n = 12	2.7 (1.1–29.7) n = 12	1.5 (0.8–3.7) n = 9
**FoxP3 median foldchange (range)**	0.8 (0.6–1.5) n = 12	1.5 (0.7–2.5) n = 9	0.9 (0.7–3.3) n = 10
**MIG median foldchange (range)**	3.4 (0.2–7.7) n = 12	7.9 (0.6–25.4) n = 9	1.4 (0.3–11.2) n = 10
**IL-10 median foldchange (range)**	0.8 (0.2–2.6) n = 11	0.6 (0.1–1.8) n = 8	(0.6–2.0) n = 11
**TGF-β1 median foldchange (range)**	1.1 (0.5–1.8) n = 12	(0.0–1.8) n = 11	1.1 (0.8–2.2) n = 12

Day 0 =  baseline prior to vaccination.

Vac+7 =  seven days after the final vaccination.

Vac+28 = 28 days after the final vaccination.

In the CS stimulated PBMC the only gene with a significant median increase in expression following vaccination was IFN-γ, *P* = 0.04 (Figure 1A). MIG, FoxP3 and IL-10 were non-significantly increased at the 7 day post vaccination time point (Figure 1B, D, and E) and TGF-β1 appeared unchanged (Figure 1C). In the unstimulated PBMC there was no significant change in the median expression of any gene studied (Figure 2). We have previously shown that in this clinical trial neither IFN-γ, measured by ex vivo ELISPOT, nor anti-CS IgG antibodies correlated with protection against malaria in a sporozoite challenge [17]. In our challenge studies volunteers are closely followed and daily blood films are taken for 21 days following sporozoite challenge when sterile protection is assumed. We are therefore able to identify the day upon which a challenged volunteer becomes blood film positive and to assess whether the vaccine group have a delay in the development of detectable parasitemia when compared to the control group. As we follow volunteers for a maximum of 21 days, volunteers with sterile protection are assigned a delay to parasitemia of 21 days for statistical analysis. In the current study, When mRNA data was examined for correlation with delay to parasitemia, we found that MIG mRNA in unstimulated PBMC at the 7 day post vaccination time point correlated with protection from sporozoite challenge (r = 0.94 *P* = 0.005, Figure 2F). A similar trend was seen in CS stimulated PBMC, although this did not reach statistical significance (r = .794 *P* = .059, Figure 1F).

The gene with the greatest fold change increase in expression both before and following vaccination was MIG, followed by IFN-γ then FoxP3 (Table 1). There was no fold increase in expression of TGF-β1 or IL-10. The fold change in expression of none of these cytokines showed any correlation with protection against malaria in the sporozoite challenge.

### Correlation of gene expression with IFN-γ ex vivo ELISPOT and anti-CS IgG antibody responses

There was no correlation of the day 7 IFN-γ ELISPOT response with MIG mRNA expression in either CS stimulated or unstimulated PBMC and no correlation of IFN-γ ELISPOT with protection from malaria. None of the cytokines measured by RT-PCR in CS stimulated PBMC correlated with the IFN-γ ELISPOT.

**Figure 3:**
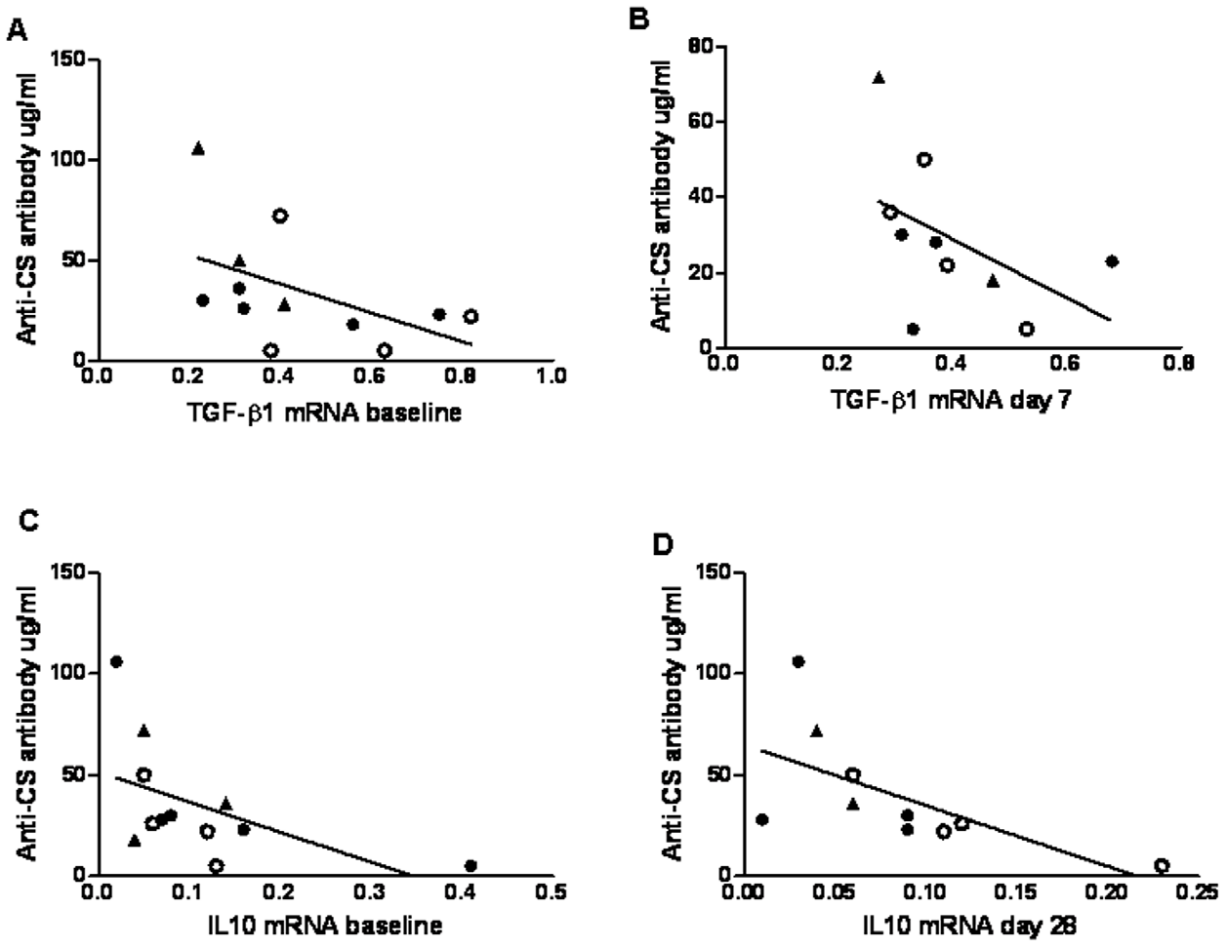
Inverse Correlation between Anti-inflammatory Cytokines and Antibody Response on the Day of Malaria Challenge. The anti-CS IgG antibody response on the day of malaria challenge induced by vaccination with RTS,S and MVA-CS inversely correlates with the anti-inflammatory cytokines TGF-β1 and IL-10 measured in unstimulated PBMC. A) TGF-β1 at baseline inversely correlates with anti-CS IgG measured on day of challenge r = −0.644 *P* = 0.022. B) TGF-β1 at day 7 inversely correlates with anti-CS IgG measured on day of challenge, r = −0.670 *P* = 0.009. C) IL-10 mRNA at baseline inversely correlates with anti-CS IgG measured on day of challenge r  = −0.554 *P* = 0.031. D) IL-10 mRNA at day 28 inversely correlates with anti-CS IgG measured on day of challenge r = −0.762 *P* = 0.005. The volunteers with sterile protection are indicated by open circles and triangles indicate volunteers who did not enter the challenge study. Correlations were performed using Spearman's two-tailed test, n = 9–12.

There was an inverse relationship between TGF-β1 mRNA in unstimulated PBMC at baseline and the anti-CS IgG antibody response measured on the day of malaria challenge r = −0.644 *P* = 0.022 (Figure 3A). TGF-β1 mRNA at day 7 also correlated with anti-CS IgG antibodies on the day of malaria challenge, r = −0.670 *P* = 0.009 (Figure 3B). An inverse relationship was also seen for IL-10 at baseline and anti-CS IgG antibody on the day of challenge in unstimulated PBMC r = −0.554 *P* = 0.031 (Figure 3C), and similarly for IL-10 at day 28 and anti-CS IgG, r = −.762 P = .005 (Figure 3D).

### Correlation between MIG and pro- and anti-inflammatory cytokine mRNA expression

**Figure 4:**
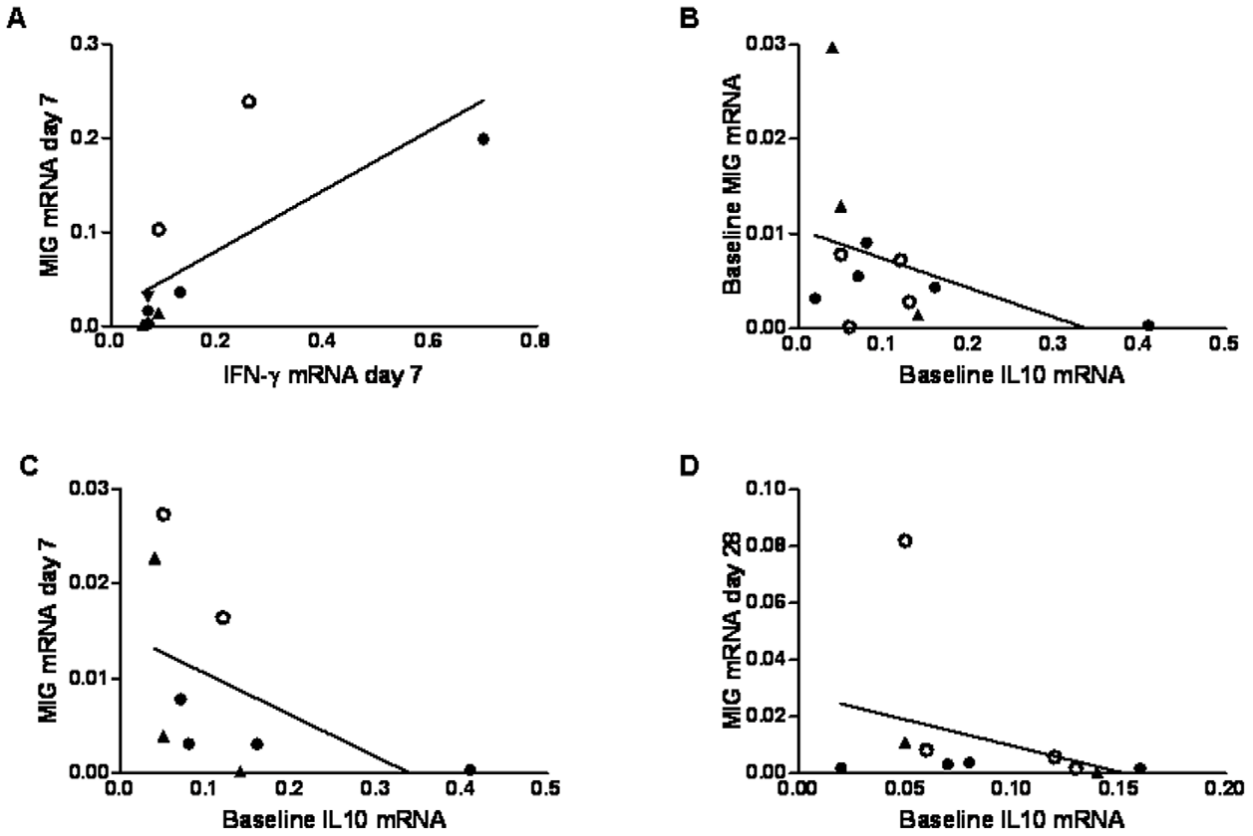
The Correlation of MIG mRNA with Pro- and Anti-inflammatory Cytokines. A) In CS stimulated PBMC the expression of MIG mRNA at day 7 correlated with the expression of IFN-g mRNA at day 7 r = 0.851 *P* = 0.004. In unstimulated PBMC IL-10 mRNA at baseline inversely correlated with B) baseline MIG mRNA r = −0.497 *P* = 0.05. C) Day 7 MIG mRNA, r = −0.787 *P* = 0.006. D) Day 28 MIG mRNA, r = −0.657 P = 0.02. The volunteers with sterile protection are indicated by open circles and triangles indicate volunteers who did not enter the challenge study. Correlations were performed using Spearman's test, n = 9–12.

As MIG mRNA was associated with protection from malaria we searched for correlations between MIG mRNA and the remaining cytokines in our data set. In the CS stimulated PBMC the expression of IFN-γ mRNA correlated with the expression of MIG mRNA, r = 0.851 *P* = 0.004 (Figure 4A). For this analysis samples were available from only two of the four volunteers with sterile protection. Both of the volunteers with sterile protection had a higher proportion of MIG mRNA compared to IFN-γ mRNA.

In the unstimulated PBMC the only cytokine to correlate with MIG expression was IL-10. There was an inverse relationship between the expression of IL-10 at baseline and MIG at baseline (Figure 4B), day 7 (Figure 4C) and day 28 (Figure 4D). At the day 7 timepoint (Figure 4C) both of the volunteers with sterile protection had a higher proportion of MIG mRNA when compared to IL-10 mRNA. Although the numbers are small these results indicate that MIG expression may be influenced by both IFN-γ and IL-10 and that high MIG expression may be a marker of sterile protection.

### Correlation of IL-10 with FoxP3 and TGF-β1 mRNA expression

**Figure 5:**
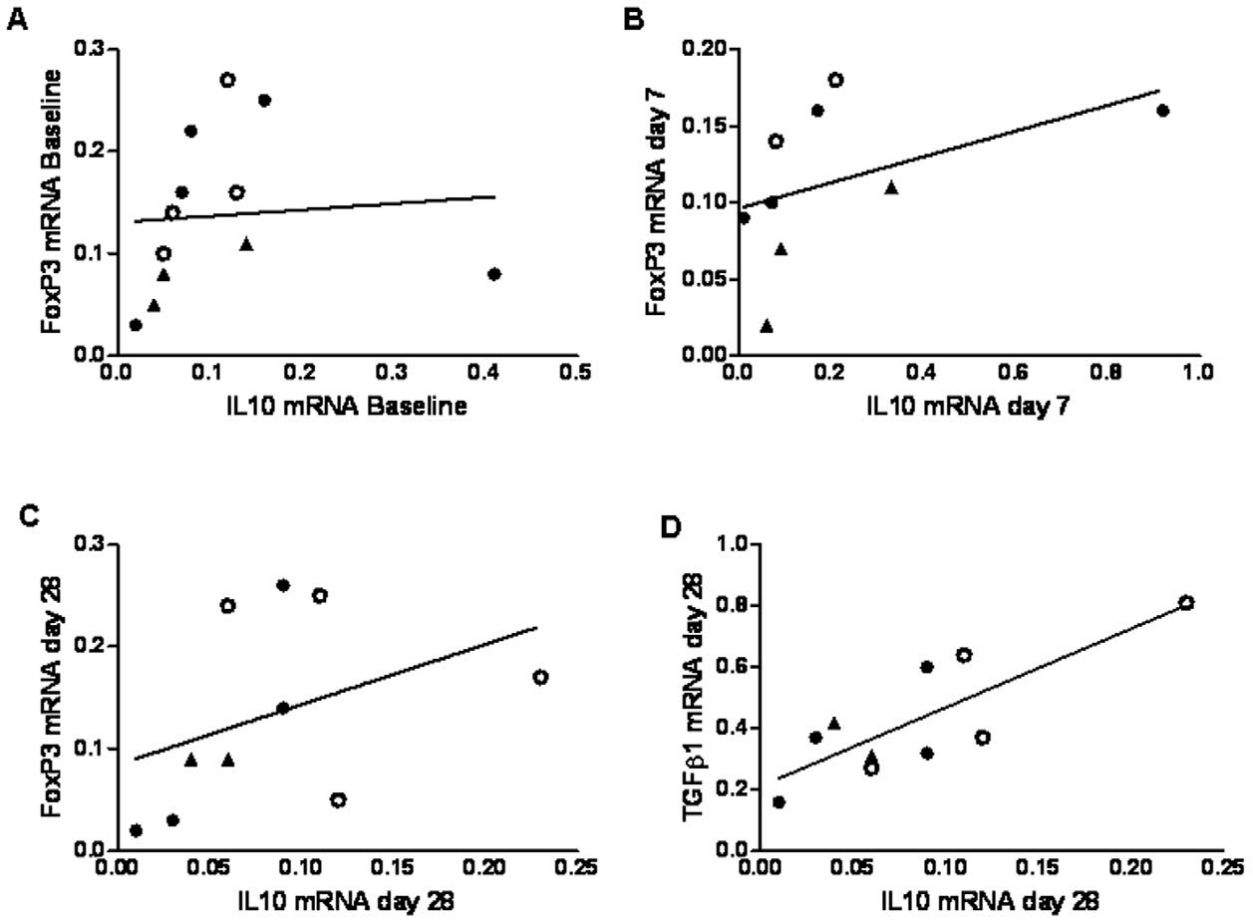
The Correlation of IL-10 expression with FoxP3 and TGFβ1 mRNA Expression. For the A) baseline (r = 0.557 *P* = 0.030), b) 7 days following vaccination (r = 0.695 P = 0.019) and C) 28 days following vaccination time points (r = 0.554 *P* = 0.048) IL-10mRNA expression correlates with FoxP3 expression. At the 28 days time point D) IL-10 mRNA also correlates with TGF-β1 mRNA expression (r = 0.642 *P* = 0.023). The volunteers with sterile protection are indicated by open circles and triangles indicate volunteers who did not enter the challenge study. Correlations were performed using Spearman's one-sided test, n = 9–12.

Although in this study there were insufficient cells to confirm the presence of regulatory T cells by flow cytometry analysis and cell depletion studies we were able to look for associations between the anti-inflammatory cytokines IL-10 and TGF-β1 and the regulatory T cell transcription factor, FoxP3. IL-10 expression correlated with FoxP3 expression in unstimulated cells in all time points tested (Figure 5A, B and C). IL-10 expression also correlated with TGF-β1 at day 28 following vaccination with RTS,S and MVA-CS.

## Discussion

Malaria vaccine development is at an exciting stage with both antibody-targeted and T cell-targeted pre-erythrocytic vaccines showing partial protection in humans [20]. However, frustratingly the most effective vaccine candidate RTS,S still only shows about 50% efficacy in the most successful phase IIa efficacy trials [21], and varying levels of protection in IIb field trials [16]. A better understanding of how some vaccine recipients are better protected than others could be crucial to developing a higher efficacy vaccine. Real time RT-PCR is an emerging method for measuring both pro-inflammatory and anti-inflammatory immune responses in humans and shows real potential for the monitoring of vaccine trials where cell numbers are limited and immune responses are often low. Using real time RT-PCR to monitor changes in gene expression in stored samples from volunteers vaccinated with RTS,S/AS02A and MVA-CS we have found that mRNA expression of pro and anti-inflammatory cytokine responses in unstimulated PBMC are associated with vaccine immunogenicity and protection from malaria in a sporozoite challenge model. The associations we have found are strongest in unstimulated PBMC and in the timepoint seven days following vaccination.

Previous studies have reported MIG detection to be a more sensitive measure of immunogenicity than the measurement IFN-γ by ELISPOT, ELISA or flow cytometry [19], [22], [23]. MIG has also been shown to be important for protection from *Trypanosoma cruzi* infection in mice [24] and is associated with disease severity in human tuberculosis [25]. MIG is induced by IFN-γ and mediated via the JAK-STAT signalling pathway [26] and is therefore a marker of bioactive IFN-γ and functional JAK-STAT signalling. In CS stimulated PBMC there was a correlation between MIG and IFN-γ mRNA, although in the two volunteers with sterile protection there was more MIG relative to IFN-γ. This may indicate either higher levels of bioactive IFN-γ or greater JAK-STAT signalling in the protected volunteers when compared to the rest of the challenge group. IL-10 is an anti-inflammatory cytokine with the primary function of regulating immune responses by activation of the macrophage JAK-STAT pathway [27]. Activation of this pathway through the IFN-γ receptor is pro-inflammatory and leads to the expression of IFN-γ induced genes, including MIG, whereas activation through the IL-10 receptor leads to immune regulation. We saw a reciprocal relationship between the expression of MIG and IL-10 mRNA at all time points studied and have found that MIG expression 7 days following final vaccination correlated inversely with time to detection of parasites by blood film in a human sporozoite challenge model. The correlation of MIG with delay to blood film positivity supports the hypothesis that T cells and bioavailable IFN-γ immune responses are important in host defence against the parasite, with previous studies demonstrating the correlation of MIG and bioavailable IFN-γ in humans as detected by RT-PCR and flow cytometry [18], [19].

Although in our study anti-CS IgG antibodies did not correlate with protection from disease, immune protection from malaria is complex and T cells as well as antibodies have been shown to be important [1], [5], [21], [28], [29]. There was no evidence that the addition of MVA-CS to the RTS,S/AS02A regimen enhanced the efficacy of RTS,S/AS02A. RTS,S/AS02A is a known powerful inducer of an antibody response [30], [31] and analysis of the immune responses from subjects in this study showed a strong antibody response and only a modest T-cell responses [17]. We have found that both IL-10 and TGF-β1 mRNA inversely correlate with the levels of anti-CS IgG antibodies following vaccination with RTS,S and MVA-CS. TGF-β1 is a peptide with pleiotropic effects on inflammation and immunoregulation and is a potent inhibitor of B cell maturation, proliferation, IgM and IgG production in the mouse [32], [33] and has also been shown to inhibit IgG production in humans [34], [35]. TGF-β1 has also been demonstrated to play a key role in the induction and maintenance of peripheral regulatory T cells in humans [36]–[38]. The inverse relationship found between TGF-β1 levels and antibody response on day of challenge is of interest. Exposure to enteric bacteria is likely to result in the development of regulatory lymphocytes as proposed in the hygiene hypothesis [39] and children with food allergy have reduced TGF-β+ T-cells in the duodenal mucosa [40]. There is also evidence of a strong genetic contribution to circulating TGF-β1 levels [41]. There is a wide variation in TGF-β levels within and between populations, for example levels of duodenal TGF-β+cells in rural Gambian infants are up to ten times higher than in UK controls [42]. We hypothesise that levels of TGF-β may be related to immune responses to vaccination. In a murine model of malaria a relationship between response to vaccination, gut parasite infestation and TGF-β1 levels has been reported [43]. Parallel studies in our laboratory have demonstrated down-regulation of TGF-β1 and increased IFN-γ ELISPOT responses following boosting of BCG vaccinated subjects with the novel tuberculosis vaccine MVA-85A [44]. Due to limited cell numbers we were unable to confirm if regulatory T cells were influencing the vaccine induced immune response or protection from disease.

Although protection induced by vaccination with RTS,S is partial it remains the best performing candidate malaria vaccine in the world. There has been no immune correlate of protection identified for RTS,S to date although both antibodies, and possibly also T cells, are thought to be important for protection [10]–[13], [45]. Our results support the view that a functional IFN-γ immune response is important for protection induced by RTS,S although whether this would work by a direct effect of cellular immunity at the liver-stage or by modulating the quality of protective antibodies induced remains unclear. The role of the MVA-CS vaccine cannot be fully ascertained in this study. MVA-CS neither induced nor boosted antibody responses and there was no evidence of improvement in efficacy compared to RTS,S used alone in other studies [17]. IL-10 and TGF-β1 may play a dual role in the attenuation of both protective T cell and IgG antibody responses induced by vaccination [46], and suggest pathways for the next generation of vaccines to target to enhance responses.

This study was based on mRNA measurement in relatively small cell numbers, giving potential for development of monitoring assays using fingerprick blood samples suitable for field trials. An immune correlate of protection would greatly facilitate the development and testing of new malaria vaccines. Our findings, in such a small dataset of twelve subjects, need to be confirmed in a larger challenge study cohort and in a field setting and more detailed analysis of the pathways involved is required. In particular the impact of baseline IL-10 and TGF-beta levels on the induction of antibodies in African populations could be assessed by monitoring volunteer samples collected prior to vaccination with RTS,S and other candidate malaria vaccines. The feasibility of mRNA profiling to assess immune responses in an African vaccine trial has been demonstrated [47]. Factors affecting the development of protective immune responses following vaccination with RTS,S/AS02A are of considerable interest to the vaccine community as further elucidation of these mechanisms could hold the key to understanding why some individuals acquire effective immunity after vaccination to life-threatening infections while others remain devastatingly vulnerable, opening doors to designing the next generation of highly effective malaria vaccines.

## Materials and Methods

### Clinical Trial

Eighteen healthy adult malaria-naïve volunteers in Oxford, UK participated in a vaccine trial as described previously [17]. The study received ethical approval from the Oxfordshire Research Ethics Committee and the Human Subjects' Protection Committee at PATH (Program for Appropriate Technology in Health) in Seattle, WA, USA. All participants gave written, informed consent prior to participation. The trial was conducted according to Good Clinical Practice guidelines, was externally monitored, and was approved by the UK Medicines and Healthcare products Regulatory Agency (MHRA). Subjects received two doses of the RTS,S/AS02A vaccine (GSK Biologicals, Rixensart, Belgium) and one dose of MVA-CS (Oxford University, Oxford, UK). 28 days after the final immunisation the efficacy of the vaccine schedule was assessed in twelve of the volunteers by experimental sporozoite challenge, whereby the volunteers were exposed to the bites of five laboratory-reared mosquitoes infected with the chloroquine-sensitive 3D7 strain of *Plasmodium falciparum*. In this study four out of twelve vaccinated subjects demonstrated complete (sterile) protection against clinical malaria (no parasitemia detectable within 21 days of challenge). For the twelve subjects as a group number of days to parasitemia was used to assess partial or complete efficacy against malaria.

### Antibody monitoring

Anti-CS repeat region antibody concentrations were measured in serum at the Walter Reed Army Institute of Research by Dr. V. Ann Stewart by specific IgG ELISA to a recombinant protein containing 32 *P. falciparum* derived tetrapeptide repeat sequences MDP-[(NANP)15NDVP]2LR [48] and expressed as GMCs (Geometric Mean Concentrations) in µg/ml. Antibodies were measured at baseline, 28 days after each vaccine dose, and three months after the malaria challenge.

### PBMC separation

Peripheral blood mononuclear cells (PBMC) were isolated by density gradient, using Lymphoprep (*Axis-Shield, Oslo, Norway*), resuspended in culture media comprising RPMI 1640 (*Sigma-Aldrich, Poole, Dorset, UK*) with 10% heat-inactivated normal human AB sera (*UK Blood Bank Service, National Health Service, UK*), 1000 U/ml penicillin-streptomycin and 2 mM L-glutamine (both *Invitrogen Life Technologies, Paisley, UK*) and stored in 10% DMSO-Foetal Calf Serum (both *Sigma-Aldrich, Poole, Dorset, UK*) in liquid nitrogen until required.

### Cell stimulation for gene expression studies

Cells for gene expression studies by RT-PCR were stimulated with one peptide pool (consisting of 61 separate 15-mer peptides designed to span the CS protein) for 12 hours overnight at a concentration of 2 µg/ml. For each sample, 1 million cells in 100 µl were plated out in each of two wells of a 96-well U-bottomed plate and incubated at 37°C for 5 hours prior to stimulation. This 5 hour resting period prior to stimulation had been shown to produce optimal results in terms of RNA yield and antigen-specific responses. 100 µl of either peptide pool or media was then added to each well to commence the 12 hour stimulation at 37°C.

### RNA extraction and RT-PCR

RNA extraction and reverse transcription of RNA into cDNA were performed using the RNeasy Mini-kit and the Omniscript kit (both *Qiagen, Crawley, West Sussex, UK*) according to the manufacturer's instructions. Quantitative real time Reverse Transcription PCR was performed using the Lightcycler 2.0 (*Roche, Basel, Switzerland*) carousel-based system using Quantitect SYBR Green Mastermix (*Qiagen, Crawley, West Sussex, UK*). All reactions were performed in duplicate with two negative controls per run. Samples were run using the Lightcycler programme, with a 15-minute incubation at 95°C followed by 45 thermal cycles, consisting of a 15-second denaturation step at 94°C, then a 20-second annealing step at 60°C, and a 20-second extension step at 72°C after which fluorescence was read. Data were produced as amplification plots with fluorescence plotted against number of cycles. The C_T_ (threshold cycle) value for each sample was calculated with the threshold set during the log-linear phase of amplification using the “Fit points” method, where the noiseband for each experiment was manually adjusted to 0.1 fluorescence units. A melt curve programme was performed at the end of each to check the melting points of the products detected for identification and specificity.

The primer sequences used were TGF-β1 F: 5′-GGACATCAACGGGTTCACTA-3′, TGF-β1 R: 5′-CCGGTTCATGCCATGAATGG-3′, IFN-γ F: 5′-ATTCGGTAACTGACTTGAATGTCC-3′, IFN-γ R: 5′-CTCTTCGACCTCGAAACAGC-3′, MIG F: 5′-GCATCATCTTGCTGGTTCTGATTGG-3′, MIG R: 5′-GCGACCCTTTCTCACTACTGGGGT-3′, FoxP3 F: 5′-CACTTACAGGCACTCCTCCAGG-3′, FoxP3 R: 5′-CCACCGTTGAGAGCTGGTGCAT-3′, IL-10 F: 5′-GGCCGTGGAGCAGGT-3′ and IL-10 R: 5′-CACTCATGG CTTTGTAGATGCC-3′.

### Statistical Analysis

The number of subjects included in this exploratory study is small. Significant findings, while useful for observing trends in the data, are prone to error and must be confirmed in larger studies. Real time RT-PCR data were interpreted using standard curves derived for each gene. To normalise for cell number, the copy number for each gene of interest was expressed as a ratio relative to the copy number of the housekeeping gene HPRT for that sample. As the data were not normally distributed non-parametric tests were used. For analysis of difference between two related samples Wilcoxon's signed rank test for significance was used. For testing of significance of correlations the two-tailed Spearman's Rank test was used unless otherwise stated. A *P* value of less than 0.05 was considered significant. The data was represented graphically using GraphPad Prism version 4 software to plot best-fit linear regression lines.
